# Potential Benefits of Nobiletin, A Citrus Flavonoid, against Alzheimer’s Disease and Parkinson’s Disease

**DOI:** 10.3390/ijms20143380

**Published:** 2019-07-10

**Authors:** Akira Nakajima, Yasushi Ohizumi

**Affiliations:** 1 Department of Applied Biology and Food Sciences, Faculty of Agriculture and Life Science, Hirosaki University, 3 Bunkyo-cho, Hirosaki 036-8561, Japan; 2 Kansei Fukushi Research Institute, Tohoku Fukushi University, 6-149-1 Kunimigaoka, Aoba-ku, Sendai 989-3201, Japan

**Keywords:** nobiletin, Alzheimer’s disease, Parkinson’s disease, neuroprotection

## Abstract

Alzheimer’s disease (AD), which is characterized by the presence of amyloid-β (Aβ) plaques and neurofibrillary tangles, accompanied by neurodegeneration, is the most common form of age-related neurodegenerative disease. Parkinson’s disease (PD) is the second most common neurodegenerative disease after AD, and is characterized by early prominent loss of dopaminergic neurons in the substantia nigra pars compacta. As currently available treatments are not able to significantly alter the progression of these diseases, successful therapeutic and preventive interventions are strongly needed. In the course of our survey of substances from natural resources having anti-dementia and neuroprotective activity, we found nobiletin, a polymethoxylated flavone from the peel of Citrus depressa. Nobiletin improved cognitive deficits and the pathological features of AD, such as Aβ pathology, hyperphosphorylation of tau, and oxidative stress, in animal models of AD. In addition, nobiletin improved motor and cognitive deficits in PD animal models. These observations suggest that nobiletin has the potential to become a novel drug for the treatment and prevention of neurodegenerative diseases such as AD and PD.

## 1. Introduction

The most common form of age-related neurodegenerative disease is Alzheimer’s disease (AD), while the second one after AD is Parkinson’s disease (PD). As currently available treatments are not able to significantly alter the progression of these diseases, successful therapeutic and preventive interventions are strongly needed.

**Figure 1:**
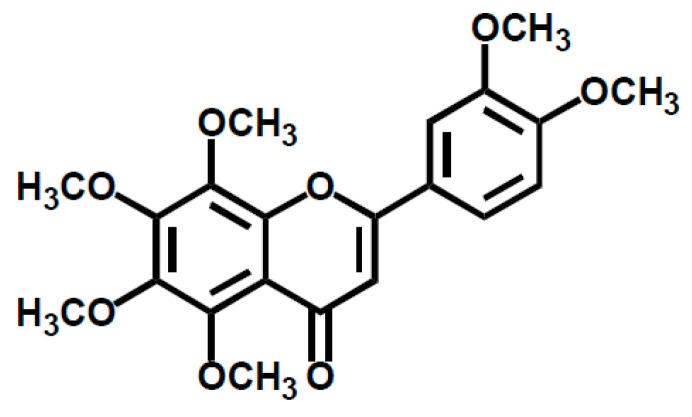
Chemical structure of nobiletin.

**Figure 2:**
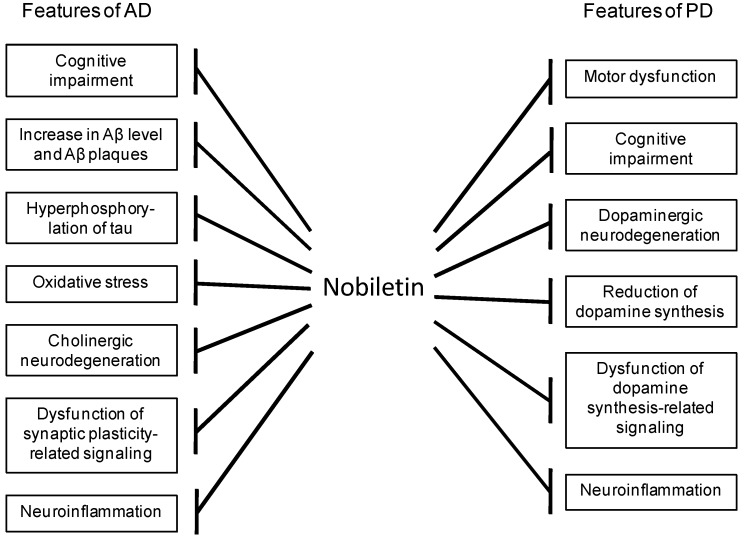
The wide range of beneficial effects of nobiletin against the features of Alzheimer’s disease (AD) and Parkinson’s disease (PD).

Nobiletin, 3′,4′,5,6,7,8-hexamethoxyflavone, is a major component of polymethoxylated flavones in the peels of citrus fruits such as Citrus depressa, Citrus reticulata, Citrus sinensis, and Citrus limon [1,2] (Figure 1). Recently, nobiletin has attracted great attention because of its beneficial health properties such as its anti-carcinogenic [3,4,5,6], anti-inflammatory [7,8,9,10], anti-atherogenic [11], anti-diabetic [12], and anti-obesity activity [13,14]. Of note, we and others have demonstrated the anti-dementia and neuroprotective properties of nobiletin through its wide range of beneficial effects, as shown in Figure 2. For example, nobiletin exhibited memory-improving effects in animal models of AD. In addition, nobiletin treatment improved motor and cognitive deficits in 1-methyl-4-phenyl-1,2,3,6-tetrahydropyridine (MPTP)-induced PD model mice. These observations suggest that nobiletin has the potential to become a novel drug for the treatment and prevention of neurodegenerative diseases such as AD and PD. The aim of this review is to summarize recent findings regarding the beneficial properties of nobiletin, including its mechanism of action against AD and PD.

A comprehensive literature search of the PubMed database was conducted for studies published through June of 2019 with multiple search terms related to the effects of nobiletin on AD and PD. Studies in in vivo animal models and in vitro cultured systems and a clinical study were collected.

## 2. Effects of Nobiletin in Animal Models of AD

The number of patients with dementia worldwide was 46.8 million in 2015, and it is estimated to increase to 131.5 million by 2050 [15]. AD is the most common type of dementia. The pathological hallmarks of AD are amyloid-β (Aβ) plaques, neurofibrillary tangles, and neuronal loss. We and others have demonstrated the beneficial effects of nobiletin against cognitive impairment and the pathological features of AD in animal models of AD, as described below.

### 2.1. Olfactory-Bulbectomized Mice

Olfactory-bulbectomized (OBX) animals develop symptoms similar to those observed in AD. They display learning and memory impairment and cholinergic neurodedegeneration in the central nervous system [16,17,18]. We examined whether nobiletin exerts beneficial effects against the features of AD in OBX mice [19,20]. Administration of nobiletin (50 mg/kg, i.p. or 50–100 mg/kg, p.o.) from postoperative day 3 for 11 days significantly improved OBX-induced short-term memory impairment in the Y-maze test [19]. OBX-induced impairment of associative memory was also improved by nobiletin treatment in the step-through passive avoidance test [19,20]. Furthermore, successive administration of nobiletin rescued the OBX-induced decrease in the acetylcholinesterase (AChE)-positive fiber density in the hippocampus by 19–32% [20]. Together, these results suggest that nobiletin improves memory impairment in OBX mice by protecting the cholinergic innervation of the hippocampus.

### 2.2. Aβ-infused Rats

Based on the amyloid hypothesis [21] that proposes an early imbalance between the production and clearance of Aβ and its accumulation, which is often the initiating factor in AD, Aβ-infused rats have been widely used as an AD model [22]. The effects of nobiletin on reference and working memory impairment in the eight-arm radial maze test were examined in rats chronically infused with Aβ1-40 into the cerebral ventricle [23]. Nobiletin (10–50 mg/kg, i.p.) was administered daily to Aβ-infused rats for 7 days before and after surgery. Reference and working memory impairment in Aβ-infused rats was improved by nobiletin treatment, suggesting that nobiletin has the ability to protect against Aβ-induced memory deterioration.

### 2.3. MK-801-Treated Mice

Dysfunctional *N*-methyl-D-aspartate (NMDA) receptor-mediated neurotransmission have been reported in behavioral changes and cognitive deficits in AD as well as depressive disorders and suicidal behavior [24,25,26,27]. To clarify the effects of nobiletin on cognitive impairment associated with NMDA receptor hypofunction, we examined the effects of nobiletin on the noncompetitive NMDA receptor antagonist MK-801-induced learning and memory impairment in mice [28]. In the step-through passive avoidance test and contextual fear conditioning test, repeated treatment with nobiletin (10–50 mg/kg, i.p.) for 7 days improved MK-801-induced memory impairment through activating extracellular signal-regulated kinase (ERK) in the hippocampus. Activation of hippocampal ERK after the passive avoidance training was shown to be necessary for consolidation of resultant learning [29,30]. Of note, 4′-demethylnobiletin, a major urinary metabolite of nobiletin in rats and mice [31,32], also had beneficial effects against MK-801-induced memory impairment in mice [33], suggesting that nobiletin and its metabolite are potential therapeutic agents for AD.

### 2.4. Senescence-Accelerated Mice

The senescence-accelerated-prone mouse 8 (SAMP8) has been demonstrated to be a relevant model for AD, because it exhibits the early onset of learning and memory deficits and several pathological features of AD [22,34,35]. We examined the effects of nobiletin on age-related cognitive impairment and the pathological features of AD, such as oxidative stress and hyperphosphorylation of tau, in SAMP8 [36]. In this study, nobiletin (10–50 mg/kg, i.p.) was administered daily to SAMP8 aged 4–6 months for 1 month prior to the behavioral experiments. Based on the behavioral experiments, nobiletin improved the object recognition memory impairment in SAMP8. Context-dependent fear memory impairment in SAMP8 was also improved by nobiletin. Biochemical analyses demonstrated that nobiletin restored the decrease in the glutathione (GSH)/glutathione disulfide (GSSG) ratio, an indicator of oxidative burden, in the brain of SAMP8. Nobiletin increased the activity of intracellular antioxidant enzymes such as glutathione peroxidase and manganese-superoxide dismutase. A decrease in the protein carbonyl level, an index of protein oxidation, was also observed in the brain of nobiletin-treated SAMP8. Moreover, nobiletin reversed the increase in tau phosphorylation at Ser202 and Thr231 in the hippocampus of SAMP8. This suggests that nobiletin improves age-related cognitive impairment in SAMP8, at least in part, by reducing oxidative stress and the hyperphosphorylation of tau.

### 2.5. Amyloid Precursor Protein Tg Mice

We examined the effects of nobiletin in amyloid precursor protein (APP)-Swedish/London (SL) 7-5 Tg mice that overexpress human APP695 harboring the double SL mutations [37]. In this study, nobiletin (10 mg/kg, i.p.) was administered daily for 4 months to 9-month-old APP-SL 7-5 Tg mice, and it significantly ameliorated context-dependent fear memory impairment. Based on immunohistochemical analyses, treatment with nobiletin reduced Aβ deposition in the hippocampus of APP-SL 7-5 Tg mice. We also analyzed quantity of Aβ with ELISA system. Administration of nobiletin significantly decreased insoluble Aβ1–40 and Aβ1–42 levels in the brain. Together, long-term treatment with nobiletin reduced the amount of Aβ and inhibited formation of Aβ plaques in APP-SL 7-5 Tg mice.

### 2.6. 3XTg-AD Mice

3XTg-AD mice harbor APPswe, PS1M146V, and tauP301L transgenes [38]. These mice progressively manifest the features of AD including Aβ plaques, neurofibrillary tangles, and behavioral abnormalities [39,40,41,42,43,44,45,46]. We examined the effects of nobiletin on the behavioral abnormalities in 3XTg-AD mice by employing several behavioral tests [47]. Male 3XTg-AD mice aged 6–7 months were used in this study. Treatment of 3XTg-AD mice with nobiletin (30 mg/kg, i.p.) for 3 months improved the impairment of short-term memory and recognition memory in the Y-maze test and novel object recognition test, respectively. ELISA analyses revealed that nobiletin significantly reduced the levels of soluble Aβ1-40 in the brain of 3XTg-AD mice. Collectively, these results suggest that nobiletin improves cognitive impairment in 3XTg-AD mice, at least in part, by reducing the soluble Aβ levels in the brain.

### 2.7. Animal Models of Cerebral Ischemia

Decreased cerebral blood flow causes cognitive deficits and neuronal injury in neurodegenerative diseases, including AD. Accordingly, the beneficial effects of nobiletin in animal models of cerebral ischemia have been examined. A previous study addressed the questions of whether nobiletin has beneficial effects on cerebral ischemia-induced neuronal death in the hippocampal CA1 region and whether it improves the cerebral ischemia-induced learning and memory deficits using a bilateral common carotid arteries occlusion (BCCAO) model mouse [48]. In the step-through passive avoidance test and Y-maze test, 5-min BCCAO-induced memory impairment was improved by nobiletin administration (50 mg/kg, i.p.) for 7 consecutive days before and after brain ischemia. According to Western blot analyses, nobiletin prevented the BCCAO-induced reduction of calcium/calmodulin-dependent protein kinase II (CaMKII), microtubule-associated protein 2 (MAP2), and GluR1 protein levels in the hippocampus. Nobiletin also prevented the BCCAO-induced reduction of CaMKII autophosphorylation and ERK phosphorylation, suggesting that the activation of CaMKII and ERK signaling, at least in part, mediates improvement of BCCAO-induced learning and memory deficits by nobiletin.

The effects of nobiletin on cerebral ischemia were also examined in a rat model of permanent middle cerebral artery occlusion (p-MCAO) [49,50]. In these studies, rats were administered nobiletin (10–25 mg/kg, i.p.) once daily for 3 days prior to surgery and then received nobiletin once again immediately after surgery. Neurological deficits and brain edema were reduced by nobiletin treatment (25 mg/kg). In addition, the infarct volume was reduced (10–25 mg/kg). Immunohistochemistry, Western blotting, and reverse transcription (RT)-polymerase chain reaction (PCR) analyses revealed that nobiletin increased the activity of Akt, CREB, brain-derived neurotrophic factor (BDNF), and Bcl-2. In addition, the pMCAO-induced reduction of claudin-5, a major component of tight junction strands, was reversed by nobiletin. Regarding its anti-oxidant and anti-inflammatory effects, the expression of Nrf2, hemeoxygenase (HO)-1, superoxide dismutase (SOD)-1, and GSH were increased, whereas the levels of NF-kB, matrix metalloproteinase (MMP)-9, and malondialdehyde (MDA) were reduced. These results suggest that nobiletin protects the brain from ischemic damage via activation of the Akt/CREB and Nrf2/HO-1 pathways, downregulation of NF-kB expression, and amelioration of blood–brain barrier (BBB) permeability.

The effects of nobiletin on ischemia-reperfusion (I/R) injury were investigated in cerebral I/R model rats induced by transient middle cerebral artery occlusion (t-MCAO) [51]. In this study, nobiletin (15 mg/kg, i.v.) was administered twice at 0 and 1 h after the start of reperfusion in t-MCAO rats. Cerebral I/R induced marked brain damage in the ischemic hemisphere of the t-MCAO rat brain, whereas nobiletin significantly reduced the infarct volume and suppressed brain edema. Based on TUNEL staining and immunohistochemical analyses, nobiletin reduced apoptotic cell death and neutrophil infiltration in the cerebral I/R region. Furthermore, it ameliorated the motor deficit induced by cerebral I/R. Therefore, nobiletin is a potential neuroprotectant for the treatment of cerebral I/R injury.

## 3. Effects of Nobiletin in Animal Models of PD

PD is the second most common neurodegenerative disease after AD and affects 2–3% of those >65 years of age. The number of PD patients worldwide was 6.1 million in 2016 [52]. PD is characterized by the early loss of dopaminergic neurons in the substantia nigra pars compacta (SNpc) [53]. The mechanisms that may lead to the degeneration of dopaminergic neurons include oxidative stress, mitochondrial dysfunction, protein aggregation and misfolding, neuroinflammation, excitotoxicity, apoptosis, and loss of trophic factors [54]. The antioxidant supply, alteration of intracellular signaling pathways, and neurotrophic factors may be useful to treat and prevent this disease [55].

Yabuki et al. reported that nobiletin administration (50 mg/kg, i.p.) for 2 consecutive weeks, starting 1 day after final MPTP injection, improved motor deficits in MPTP-induced PD model mice in the rotarod test and beam-walking test [56], and improved MPTP-induced cognitive deficits in the passive avoidance test and novel object recognition test [56]. However, in immunohistochemical experiments, nobiletin did not block the loss of tyrosine hydroxylase (TH)-positive dopaminergic neurons in the ventral tegmental area (VTA) and SNpc in MPTP-treated mice. Similarly, it did not rescue the decreased TH protein levels in the striatum and hippocampal CA1 region in these mice based on Western blot analyses. In contrast, nobiletin restored the MPTP-induced decrease in CaMKII autophosphorylation and phosphorylation at Thr-34 of dopamine- and cAMP-regulated phosphoprotein-32 (DARPP-32) in the striatum and hippocampal CA1 region. Moreover, TH phosphorylation at Ser-19 and Ser-40, that are CaMKII- and PKA-dependent phosphorylation sites, was increased, suggesting that nobiletin increases TH activity by stimulating CaMKII- and PKA-dependent phosphorylation in the striatum and hippocampal CA1 region, events that likely lead to motor and memory improvement. The MPTP-induced reduction of dopamine in the striatum and hippocampal CA1 region was improved by nobiletin, which was expected because TH is the rate-limiting enzyme in dopamine synthesis [57]. Thus, nobiletin rescues motor and cognitive dysfunction in MPTP-induced PD model mice, at least in part by stimulating CaMKII and cAMP signaling, which are important intracellular signaling pathways for dopamine synthesis.

Previously, nobiletin was reported to protect dopaminergic neurons in the substantia nigra in the 1-methyl-4-phenylpyridinium (MPP^+^)-treated rat model of PD [58]. MPP^+^, a toxic metabolite of MPTP, specifically penetrates dopaminergic neurons via dopamine transporters, causing their selective degeneration [59]. Nobiletin administration (10 mg/kg, i.p.), starting 1 day before MPP^+^ injection and continuing until 6 days postinjection, significantly protected dopaminergic neurons in the substantia nigra of MPP^+^-treated rats, accompanied by the inhibition of microglial activation and preservation of the glial cell line-origin neurotrophic factor expression. These results suggest that nobiletin protects dopaminergic neurons from MPP^+^-induced toxicity, at least in part, by inhibiting neuroinflammation.

## 4. Effects of Nobiletin in Cultured Cells and Slices

To clarify the mechanism of action of nobiletin, its effects on protein kinase A (PKA)/ERK/CREB signaling, oxidative stress, endoplasmic reticulum (ER) stress, neuroinflammation, and Aβ generation and degradation were examined in cultured cells and slices, as described below. 

### 4.1. Effects of Nobiletin on the cAMP/PKA/ERK/CREB Signaling Pathway

Increases in intracellular levels of cAMP lead to the activation of PKA and subsequent phosphorylation of the transcription factor CREB. cAMP also activates Rap1, a small GTP-binding protein in the Ras family that serves as a selective activator of B-Raf [60]. The B-Raf-Rap1 complex in turn activates ERK, leading to CREB phosphorylation [60,61]. The cAMP/PKA/ERK/CREB signaling pathway plays essential roles in memory formation in vivo and in long-term potentiation (LTP), a synaptic model of memory [62,63,64,65,66].

Aβ was demonstrated to inhibit the formation of hippocampal late LTP through the inactivation of the PKA/CREB signaling pathway [67]. Aβ oligomers also inhibit ERK and CREB activation in primary hippocampal neurons [68]. Therefore, agents that enhance the cAMP/PKA/ERK/CREB signaling pathway may be useful for the treatment and prevention of AD.

Nagase et al. previously reported that nobiletin inhibits the phosphodiesterase (PDE) activity catalyzing the hydrolysis of cAMP, thereby increasing the intracellular cAMP concentration to activate PKA in PC12D cells [69]. Moreover, nobiletin induced CREB phosphorylation in an ERK kinase (MEK)/ERK-dependent manner [19,70,71]. Of note, it also reversed the sublethal Aβ-induced decrease in CREB phosphorylation in cultured hippocampal neurons [23].

Matsuzaki et al. demonstrated that nobiletin increases the phosphorylation of multiple PKA substrates and the GluR1 receptor, the subunit of α-amino-3-hydroxy-5-methyl-D-aspartate (AMPA) receptors, at the PKA phosphorylation site Ser 845 in cultured hippocampal neurons and hippocampal slices [72]. The phosphorylation of GluR1 at Ser 845 is essential for synaptic plasticity, including enhancement of the postsynaptic glutamate response [73,74]. Of note, nobiletin was found to potentiate AMPA receptor-mediated synaptic transmission at Schaffer collateral-CA1 pyramidal cell synapses in hippocampal slices [72]. This potentiation was not accompanied by changes in the paired-pulse ratio, suggesting involvement of a postsynaptic mechanism. Thus, nobiletin may upregulate synaptic transmission via postsynaptic AMPA receptors, at least in part, by stimulating PKA-mediated phosphorylation of the GluR1 receptor in the hippocampus.

In addition to the AMPA receptor, the NMDA receptor plays an important role in synaptic plasticity [75,76,77]. Aβ induces persistent depression of NMDA-evoked currents, suggesting that prolonged depression of NMDA receptor-mediated transmission functions in the initiation of the pathological changes observed in AD [78]. It has also been reported that the NMDA receptor subunits GluN1 and GluN2B mRNA and protein levels are reduced in AD patients, and the degree of reduction is associated with progression of the neuropathology [79]. Kimura et al. reported that nobiletin significantly upregulated the mRNA expression of GluN1, GluN2A, and GluN2B in PC12D cells [80]. It is well known that activation of NMDA receptors leads to an influx of Ca^2+^, which in turn stimulates numerous signaling pathways that converge on the ERK signaling cascade [81]. Collectively, the activation of the cAMP/PKA/ERK/CREB signaling pathway is a plausible mechanism by which nobiletin exerts memory-improving effects in animal models of AD.

### 4.2. Effects of Nobiletin on Oxidative Stress and ER Stress

The effects of nobiletin on oxidative stress and ER stress have been examined in cultured cells, including PC12 cells, HT22 cells, and SK-N-SH cells. It was reported to have neuroprotective effects against hydrogen peroxide (H_2_O_2_)-induced apoptosis in PC12 cells, accompanied by inhibition of H_2_O_2_-induced decrease in GSH levels and SOD activity [82]. Although nobiletin has negligible direct antioxidant activity, it induces intracellular GSH through the upregulation of glutamate-cysteine ligase, a rate-limiting enzyme for GSH synthesis, and protects against serum withdrawal- and H_2_O_2_-induced cytotoxicity in PC12 cells [83]. It was also reported to protect against H_2_O_2_-induced cell death in HT22 cells, a murine hippocampal cell line, accompanied by the inhibition of c-Jun N-terminal kinase (JNK) and p38 phosphorylation [84]. In addition, it inhibited the H_2_O_2_-induced increase in the expression of pro-apoptotic protein Bax, whereas anti-apoptotic protein Bcl-2 was induced by nobiletin in HT22 cells [84].

Nobiletin-induced alterations of gene expression have been examined by DNA microarrays in three organ-derived cell lines: 3Y1 rat fibroblasts, HuH-7 human hepatocarcinoma cells, and SK-N-SH human neuroblastoma cells [85]. Nobiletin administration resulted in greater than 200% increases in the levels of five genes, including the ER stress responsive genes Ddit3, Trib3, and Asns, in all three cell lines. It also induced greater than 50% decreases in the levels of seven genes, including the cell cycle-regulating genes Ccna2, Ccne2, and E2f8, as well as the oxidative stress-promoting gene Txnip. Increases in the levels of DDIT3 and ASNS proteins, and a decrease in the level of the TXNIP protein, were confirmed in each nobiletin-treated cell line. In addition, treatment of SK-N-SH cells with nobiletin suppressed the increase in TXNIP expression and apoptosis induced by tunicamycin, a potent revulsant of ER stress [86]. These results suggest that nobiletin can suppress oxidative stress and excessive/prolonged ER stress.

### 4.3. Effects of Nobiletin on Neuroinflammation

Neuroinflammation aids in the progression of neurodegenerative diseases, including AD and PD [87,88]. Nobiletin has been demonstrated to have anti-neuroinflammatory capacity by inhibiting the lipopolysaccharide (LPS)-induced production and secretion of proinflammatory mediators, such as NO, tumor necrosis factor (TNF)-α, interleukin (IL)-1β, and IL-6, in the mouse microglia BV-2 cell line [10,89,90]. In a recent report, nobiletin administration (100 mg/kg/day, p.o.) to mice for 6 weeks was found to ameliorate LPS-induced memory deficits, accompanied by the suppression of microglial activation and proinflammatory cytokine secretion [91]. These results suggest that nobiletin is useful to prevent or halt the progression of inflammation-related neurodegenerative diseases, including AD and PD.

### 4.4. Effects of Nobiletin on Aβ Generation and Degradation

Aβ is continuously generated and degraded in the brain [92]. Therefore, its steady-state level is determined by the balance between its generation and degradation [93]. Aβ is generated from the APP through endoproteolysis by two proteases called β- and γ-secretases. As β- and γ -secretases are necessary for Aβ generation; these enzymes are prime drug targets for reducing Aβ levels in AD [94]. A recent in vitro study demonstrated that nobiletin exhibits β-secretase (BACE1) inhibitory activity in a non-competitive manner with IC50 values of 5.9 × 10^−5^ M, suggesting it to be a promising BACE1 inhibitory agent to reduce Aβ production in AD [95].

Regarding Aβ degradation, neprilysin was identified as the major protease involved in the physiological degradation of Aβ in the brain [93], and it was found to be down-regulated in the AD brain [96]. Nobiletin was reported to increase the expression and activity of neprilysin in SK-N-SH cells [97]. Kimura et al. recently examined whether nobiletin promotes Aβ degradation using human-induced pluripotent stem (iPS) cell-derived AD model neurons, which generate excess Aβ1-42 due to the familial AD presenilin-1 mutation [98]. The AD-type iPS cell harbors the Polish familial AD mutation of presenilin-1 (i.e., P117L-presenilin-1), and consequently exhibits increased Aβ1-42 generation after differentiation into neurons. In contrast, the wild-type iPS cells have no mutation in this gene and were used as a control. According to real-time quantitative RT-PCR, neprilysin mRNA levels were significantly upregulated by nobiletin in the AD model neurons. Immunostaining with an anti-Aβ antibody demonstrated that nobiletin reduced the intraneuronal Aβ level. Furthermore, it reduced the level of Aβ1-42 released into the culture medium based on ELISA analyses. Therefore, nobiletin reduced the levels of both intra- and extracellular Aβ, likely via the upregulation of neprilysin and promotion of Aβ degradation under in vitro AD pathological conditions. Together, the regulation of APP processing and the promotion of Aβ degradation induced by nobiletin may play a role in the reduced Aβ levels observed in the brains of nobiletin-treated APP-SL 7-5 and 3XTg-AD mice.

## 5. Conclusions

In this review, we summarized the beneficial effects of nobiletin against neurodegenerative diseases, such as AD and PD, in both animal models and cell culture systems. Nobiletin displays a wide range of beneficial effects against the features of AD and PD. However, further studies are needed to determine its primary molecular targets using proteomic-based approaches.

Regarding clinical application, a pilot clinical study was conducted to investigate the feasibility and safety of cotreatment with nobiletin-rich Citrus reticulata peel extract and donepezil in donepezil-preadministered AD patients [99]. In this study, six patients with mild-to-moderate AD were enrolled for treatment with nobiletin-rich Citrus reticulata peel extract. Five mild-to-moderate AD patients who were preadministered donepezil were enrolled for donepezil treatment alone as the control during the same period. Cognitive function was assessed with the mini-mental state examination (MMSE) and the Japanese version of the Alzheimer’s Disease Assessment Scale-Cognitive Subscale (ADAS-J cog). One-year intervention with nobiletin-rich Citrus reticulata peel extract was suggested to prevent the progression of cognitive impairments in the patients, with no adverse events [99]. Further evidence from clinical trials is needed to establish the efficacy and safety of nobiletin as a useful strategy for the prevention and treatment of neurodegenerative diseases such as AD and PD.

## Abbreviations

AChE	Acetylcholinesterase
AD	Alzheimer’s disease
AMPA	α-amino-3-hydroxy-5-methyl-D-aspartate
Aβ	Amyloid-β
APP	Amyloid precursor protein
BACE1	β-secretase
BBB	Blood–brain barrier
BCCAO	Bilateral common carotid artery occlusion
BDNF	Brain-derived neurotrophic factor
CaMKII	Calcium/calmodulin-dependent protein kinase II
CREB	cAMP response element-binding protein
DARPP-32	Dopamine- and cAMP-regulated phosphoprotein-32
ER	Endoplasmic reticulum
ERK	Extracellular signal-regulated kinase
GSH	Glutathione
GSSG	Glutathione disulfide
HO	Hemeoxygenase
IL	Interleukin
iPS	Induced pluripotent stem
I/R	Ischemia-reperfusion
JNK	c-Jun N-terminal kinase
LPS	Lipopolysaccharide
LTP	Long-term potentiation
MAP2	Microtubule-associated protein 2
MDA	Malondialdehyde
MMP	Matrix metalloproteinase
MPP^+^	Matrix metalloproteinase
MPTP	1-methyl-4-phenyl-1,2,3,6-tetrahydropyridine
NMDA	*N*-methyl-D-aspartate
OBX	Olfactory-bulbectomized
PDE	Phosphodiesterase
PKA	Protein kinase A
p-MCAO	Permanent middle cerebral artery occlusion
PD	Parkinson’s disease
SAMP8	Senescence-accelerated-prone mouse 8
SNpc	Substantia nigra pars compacta
SOD	Superoxide dismutase
TH	Tyrosine hydroxylase
t-MCAO	Transient middle cerebral artery occlusion
TNF	Tumor necrosis factor
VTA	Ventral tegmental area
